# Management strategies and outcomes of acute coronary syndrome (ACS) during Covid-19 pandemic

**DOI:** 10.1186/s12872-022-02680-z

**Published:** 2022-05-25

**Authors:** Mingliang Zuo, Shoubo Xiang, Sanjib Bhattacharyya, Qiuyi Chen, Jie Zeng, Chunmei Li, Yan Deng, Chungwah Siu, Lixue Yin

**Affiliations:** 1 Department of Cardiovascular Ultrasound and Non-invasive Cardiology, Sichuan Academy of Medical Sciences and Sichuan Provincial People’s Hospital, University of Electronic Science and Technology of China, Chengdu, 610072 China; 2 West China Hospital, Sichuan University, Chengdu, China; 3 College of Pharmaceutical Sciences, Southwest University, Beibei, Chongqing, China; 4 Cardiology Division, Department of Medicine, Queen Mary Hospital, The University of Hong Kong, Room 1929, Block K, 102 Pokfulam Road, Hong Kong SAR, China; 5 Department of Cardiology, Sichuan Academy of Medical Sciences and Sichuan Provincial People’s Hospital, University of Electronic Science and Technology of China, Chengdu, China

**Keywords:** COVID-19, Acute coronary syndrome, Pandemic, Management, Outcomes

## Abstract

**Background:**

The COVID-19 outbreak represents a significant challenge to international health. Several studies have reported a substantial decrease in the number of patients attending emergency departments with acute coronary syndromes (ACS) and there has been a concomitant rise in early mortality or complications during the COVID-19 pandemic. A modified management system that emphasizes nearby treatment, safety, and protection, alongside a closer and more effective multiple discipline collaborative team was developed by our Chest Pain Center at an early stage of the pandemic. It was therefore necessary to evaluate whether the newly adopted management strategies improved the clinical outcomes of ACS patients in the early stages of the COVID-19 pandemic.

**Methods:**

Patients admitted to our Chest Pain Center from January 25th to April 30th, 2020 based on electronic data in the hospitals ACS registry, were included in the COVID-19 group. Patients admitted during the same period (25 January to 30 April) in 2019 were included in the pre-COVID-19 group. The characteristics and clinical outcomes of the ACS patients in the COVID-19 period group were compared with those of the ACS patients in the pre-COVID-19 group. Multivariate logistic regression analyses were used to identify the risk factors associated with clinical outcomes.

**Results:**

The number of patients presenting to the Chest Pain Center was reduced by 45% (*p* = 0.01) in the COVID-19 group, a total of 223 ACS patients were included in the analysis. There was a longer average delay from the onset of symptom to first medical contact (FMC) (1176.9 min vs. 625.2 min, *p* = 0.001) in the COVID-19 period group compared to the pre-COVID-19 group. Moreover, immediate percutaneous coronary intervention (PCI) (80.1% vs. 92.3%, *p* = 0.008) was performed less frequently on ACS patients in the COVID-19 group compared to the pre-COVID-19 group. However, more ACS patients received thrombolytic therapy (5.8% vs. 0.6%, *p* = 0.0052) in the COVID-19 group than observed in the pre-COVID-19 group. Interestingly, clinical outcome did not worsen in the COVID-19 group when cardiogenic shock, sustained ventricular tachycardia, ventricular fibrillation or use of mechanical circulatory support (MCS) were compared against the pre-COVID-19 group (13.5% vs. 11.6%, *p* = 0.55). Only age was independently associated with composite clinical outcomes (HR = 1.3; 95% CI 1.12–1.50, *p* = 0.003).

**Conclusion:**

This retrospective study showed that the adverse outcomes were not different during the COVID-19 pandemic compared to historical control data, suggesting that newly adopted management strategies might provide optimal care for ACS patients. Larger sample sizes and longer follow-up periods on this issue are needed in the future.

## Background

Coronavirus Disease 2019 (COVID-19) characterizes as a severe acute respiratory syndrome due to coronavirus-2 (SARS-CoV-2) first emerged in Wuhan, China in December 2019 [1–4], and rapidly spread worldwide. Several reports have demonstrated a substantial drop in the number of patients attending emergency departments with acute coronary syndromes (ACS), and concurrent increases in early mortality or complications during the COVID-19 pandemic have been observed [5–9]. Therefore, a new strategy to combine prevention with reduction of the impact on the clinical outcomes of ACS during the pandemic has become extremely important.

At present, stringent pandemic prevention measures included isolation, quarantine, social distancing, community containment and city lockdown. In order to comply with these measures, routine medical services were reduced. While effective in slowing down the spread of COVID-19, the measures have inevitably caused significant disruption and delay in the treatment of those patients with ACS. For instance, in order to prevent in-hospital COIVD-19 spread, patients had to undergo a test to determine their COVID-19 status prior to receiving more targeted therapeutic procedure. During the COVID-19 pandemic, it has been that the number of patients admitted for acute myocardial infarction (AMI) declined up to 48% with a longer duration from symptom onset to the first medical contact time, and this was associated with poor clinical outcomes [6–10].

Therefore, finding a balance between risks related to untimely treatment of ACS patients and COVID19 infection control has become a global challenge during this pandemic.

In Chengdu, China, the Chest Pain Center of Sichuan Provincial People's Hospital, formulated and adopted a new clinical management that emphasized nearby treatment, safety, and protection, alongside closer and more effective multiple collaborative teams to streamline the management of patients presenting with ACS due to the pandemic [11]. However, the impact of the new strategy on the clinical outcomes of ACS patients has not been assessed during the early pandemic. Therefore, there is an urgent need to evaluate whether the newly adopted management strategies improved clinical outcomes in patients with ACS, from the early phase through to the convalescent phase, regarding ACS management in our hospital.

## Methods

### Ethical consideration

This retrospective study was based on a single hospital registry of ACS in Sichuan Provincial People's Hospital, Chengdu, Sichuan, China. The study protocol was approved by the local Institutional Review Board. Informed consent from patients was not necessary given the registered nature of the study; nonetheless all patient records/information were anonymous prior to analysis.

### Patients

The analytical cohort for this study consisted of adults (aged ≥ 18 years old) admitted to our Chest Pain Center based on electronic data records. To compare the trends before and during the COVID-19 pandemic, patients admitted between 25th January and 30th April 2020 were defined as the ‘COVID-19’ period group, whereas a comparative group of patients hospitalised during the same period (25th January and 30th April 2019) were grouped as the ‘pre-COVID-19’ group. As further investigation was required to evaluate whether the newly adopted management strategies improved the clinical outcomes in patients with ACS, patients without an established diagnosis of ACS from our Chest Pain Center were excluded. Patients positive for COVID-19 were also excluded. ACS is caused by a critical obstruction of a coronary artery because of atherosclerotic coronary artery disease. Three specific conditions are included: ST elevation MI (STEMI), non-ST elevation myocardial infarction (NSTEMI), and unstable angina [12]. Patients with Acute Myocardial Infarction (AMI) were further classified into STEMI and NSTE-ACS [13].

### A newly modified management of ACS patients development

In pre-pandemic period, a regional STEMI care network was already established through collaboration between hospitals of different levels and emergency medical systems (EMS). That is, prehospital information of referred patients including clinical status, electrocardiograms (ECG) findings, and high-sensitive cardiac troponin I (hscTn-I) levels was launched using social media software such as WeChat or QQ from non-PCI regional hospitals. Also, dual antiplatelet (aspirin of 300 mg and clopidogrel of 600 mg with loading dose) treatment was employed according to standard guidelines, unless the risk of bleeding was high.

At the beginning of the COVID-19 pandemic, a newly modified management that emphasized nearby treatment, safety, and protection [11] was developed by our Chest Pain Center based on Chinese expert advice (first edition) regarding the diagnosis and treatment process of acute myocardial infarction in the prevention and control of coronavirus with protocols for patients with STEMI or NSTEM. Additionally, a recent influenza infection, influenza-like illness, or other respiratory tract infections were significantly more likely to occur in AMI cases [14]. Therefore, a multiple disciplinary team (MDT) was established to obtain prompt recognition, and early management under the background of COVID-19. In brief, a closer and more effective collaborative team was established compared with the pre-COVID19 period, and advanced professional support was continued on social media-based platforms such as WeChat or QQ too.

Based on the newly modified management of ACS, all patients were required to undergo the COVID-19 nucleic acid test and chest computerized tomography (CT) prior to receiving more targeted therapeutic procedures according to the adopted protocol. If within reperfusion time, and no contraindications for thrombolysis, the patients suspected or diagnosed with positive COVID were isolated and began thrombolytic therapy immediately. The outcomes of thrombolysis and the plan for elective angiogram/ PCI were reassessed afterwards. High- risk patients with contraindications for thrombolysis were assessed for their risk of infection and the benefit of PCI. PCI was only performed for culprit vessel required.

### Data collection

We developed a uniform form to collect the following information for every ACS patient: (1) the characteristics of each patient, including their gender, age, BMI, BP, smoking history, congestive cardiac failure, atrial fibrillation, previous myocardial infarction (MI), previous PCI, cerebrovascular disease or peripheral vascular disease, antiplatelet/ thrombolysis drug use status and history of diabetes and hypertension, ECG, and prehospital information including time to worsen symptoms prior to entry, route of presentation, FMC. (2) The results of laboratory and echocardiography examinations including high-sensitivity Cardiac troponin I (hscTn-I), B-type natriuretic peptide (BNP), heat-sensitive shock protein (HSP90a), left ventricular ejection fraction (LVEF) and LV regional wall-motion abnormality (present vs. absent). (3) The PCI details, including the number of coronary lesions, the culprit vessel (CV, as acute occlusion of coronary artery for AMI.), activation time of catheter, door-to-balloon (D2B), the time from arrival to puncture, the guide wire crossing time, duration of operation. (4) The primary outcomes, including in-hospital death, cardiogenic shock, sustained VT/VF and use of MCS.

### Statistical analysis

All of the statistical analyses were performed using SPSS 19.0 (SPSS Inc., Chicago, USA). Between-group comparisons were done using the Student’s t test for continuous variables and Pearson’s chi-square test or Fisher’s exact test for categorical variables. Univariable and multivariable logistic regression analysis was performed in order to identify independent factors associated with adverse outcomes. Hazard ratio (HR) with 95% confidence intervals (95% CIs) were calculated. We examined the effect of the newly adopted strategy on adverse outcomes by adjusting for traditional confounders. The multivariable logistic regression model included variables such as time to worsen symptoms prior to entry, route of presentation (transfer hospital or direct) during the COVID-19 outbreak, as well as traditional factors in ACS patients. As such, the following important covariates were included: age, gender, time to worsen symptoms prior to entry, previous MI, proportion of FMC within 2 h, CV (LAD/LCX /RCA), Group (COVID-19 /pre-COVID-19), proportion of D2B within 90 min and route of presentation (transfer or direct), which had a p value < 0.2 when univariate analysis first. A two-sided *p* value of < 0.05 was considered statistically significant.

## Results

### Changed admission during the COVID-19 pandemic

**Fig. 1:**
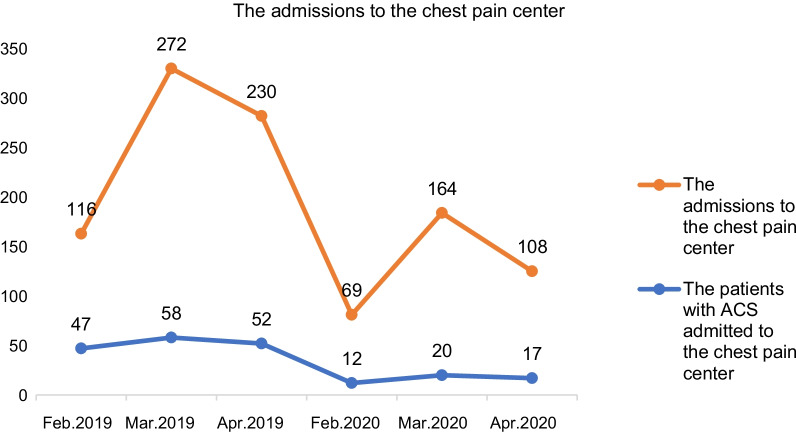
The admissions to the chest pain center. The number of patients registered in Chest Pain Center decreased from Feb. Mar. Apr. of 2019 to equivalent month in 2020 respectively. The similar trend was also found in patients with ACS

Between January 25th and April 30th 2019, the number of patients presenting to the Chest Pain Center was 630, and this was reduced by 45% (*p* = 0.01) to 347 in 2020 during the pandemic (Fig. 1). Admissions were restricted from 116 in Feb. 2019 to 69 in Feb. 2020, from 272 in Mar. 2019 to 164 in Mar. 2020 and from 230 in Apr. 2019 to 108 in Apr. 2020.

A total of 223 ACS patients (mean age 64.2 ± 13.3 years, 82.5% male) were included in the analysis, including 52 patients negative for COVID-19 in the COVID-19 group and 171 patients in the pre-COVID-19 group. Of these, 71.1% (37/52) of ACS patients in 2020 were transferred to our center via a regional non-PCI-capable facility, at a higher rate than that in 2019 (61.4%, 105/171). And the population in each month in 2020 was decreased compared with that in 2019 (Fig. 1).

### The baseline characteristics of the ACS patients within the study

The baseline characteristics of the 223 AMI patients are presented in Table 1. There were no significant differences relating to age, gender, BMI, blood pressure, history of smoking, prevalence of hypertension (HT), diabetic mellitus (DM), congestive cardiac failure, atrial fibrillation, previous MI, previous PCI, cerebrovascular disease or peripheral vascular disease between the two groups (Table 1). There were more patients transferred with ACS in the COVID-19 group than that in the pre-COVID group (*p* = 0.04).

**Table 1:** Baseline characteristics and delays of included patients

Variables	COVID-19 group (n = 171)	pre-COVID-19 group (n = 52)	*p*
Age (yrs)	64.0 ± 13.1	64.4 ± 13.5	0.85
Gender (male), n (%)	142 (83.0)	42 (80.7)	0.77
BMI (kg/m^2^)	21.5 ± 3.2	22.3 ± 4.0	0.81
SBP (mmHg)	124.60 ± 25.12	127.72 ± 30.90	0.44
DBP (mmHg)	78.59 ± 17.22	80.21 ± 18.62	0.55
Hypertension, n (%)	87 (50.9)	26 (50.0)	0.88
Diabetes mellitus, n (%)	36 (21.1)	12 (23.1)	0.12
Smoker, n (%)	110 (64.3)	34 (65.4)	0.87
Congestive cardiac failure, n (%)	2 (1.1)	1(1.9)	0.68
Atrial fibrillation, n (%)	3 (1.7)	2 (3.8)	0.39
Previous MI, n (%)	12 (7.0)	5(9.6)	0.48
Previous PCI, n (%)	8 (5.3)	3 (5.8)	0.86
Cerebrovascular disease or peripheral vascular disease, n (%)	20 (1.7)	8 (15.3)	0.66
Average FMC, min	625.3	1176.9	0.001*
Proportion of FMC within 2 h, n (%)	51 (29.8)	14 (26.9)	0.57
Time to worsen symptoms prior to entry (hrs)	10.15 ± 9.04	11.63 ± 10.61	0.68
Proportion of transferred patients, n (%)	105 (61.4)	37 (71.1)	0.04*
Catheter activation, min	5.29 ± 11.86	4.55 ± 7.01	0.65
Average D2B, min	84.3	107.1	0.28
Proportion of D2B within 90 min, n (%)	106 (61.9)	24 (46.1)	0.04*
The time from arrival to puncture, min	10.3 ± 7.8	12.9 ± 11.8	0.21
The guide wire crossing time, min	28.5 ± 11.4	21.9 ± 9.4	0.02*
Duration of operation, min	52.9 ± 22.7	44.0 ± 19.6	0.02*

*Statistical analysis was done with Student’s t test or Chi-square test, where* p* < 0.05 was considered as significant

### PCI details

According to the location of the culprit artery, the proportion of left main trunk (LMT) and left ascending branch (LAD) as culprit lesion was highest (47.5%, 106/223), 37.2% (83/223) of right coronary artery (RCA) and 15.2% (34/223) of left circumflex artery (LCX) in all ACS patients. We found similar ratio of location distribution with culprit lesion between the two groups, 46.1% (24/52) in LMT/LAD, 36.5% (19/52) in RCA and 15.4% (8/52) in LCX in the COVID-19 group, compared with that of ratio with 47.4% (81/171) in LMT / LAD, 37.4% (64/171) in RCA and 15.2% (26/171) in LCX in the pre-COVID19 group.

No significant difference was observed on the proportion of FMC within 2 h (26.9%, 14/52 vs. 29.8%, 51/171), time to worsen symptoms prior to entry between the two groups. However, average FMC was significantly different between the two groups (1176.9 min in the COVID-19 group vs. 625.2 min in the pre-COVID-19 group, respectively, *p* = 0.001). The average D2B was 23 min longer in the COVID-19 group compared with that in the pre-COVID19 group but didn’t reach the significance (*p* = 0.28). The proportion of patients with D2B within 90 min was lower in the COVID-19 group compared with that in the pre-COVID19 group (61.9%, 106/171 vs. 46.1%, 24/52, *p* = 0.04) (Table 1). The guide wire crossing time in the COVID-19 group was shorter than that observed in the pre-COVID19 group (21.9 ± 9.4 min vs. 28.5 ± 11.4 min, respectively; *p* = 0.02). A similar shorter duration of the operation was observed between the two groups (44.0 ± 19.6 min in the COVID-19 group vs. 52.9 ± 22.7 min in the pre-COVID19 group, respectively; *p* = 0.02).

### Examinations and clinical outcomes

A greater population of patients received dual antiplatelet therapy in the COVID-19 group (73.1%, 38/52) than that recorded in the pre-COVID19 group (56.1%, 96/171; *p* = 0.03). Moreover, 5.8% (3/52) of the ACS patients received thrombolytic therapy in the COVID-19 group versus 0.6% (1/171) within the ACS patients in the pre-COVID19 group (*p* = 0.0052). The proportion of immediate PCI performed in the COVID-19 group (92.3%, 48/52) was also lower than that in the pre-COVID19 group (80.1%, 137/171; *p* = 0.008).

No significant differences in the peaks for hscTn-I, BNP, LVEF and proportion of abnormal segments were present between the two groups. Only the level of HSP90a in the COVID-19 group was higher than that in the pre-COVID group (156.5 ± 106.3 vs. 104.2 ± 71.8, *p* = 0.004) (Table 2).

**Table 2:** The changes in the clinical process and composite outcomes of patients before and after COVID-19 pandemic

Variables	COVID-19 group (n = 171)	pre-COVID-19 group (n = 52)	*p*
Peak hscTn-I, ng/L	63,374.6 ± 12,261.8	111,995.2 ± 2467.3	0.18
Peak of BNP, pg/ml	648.5 ± 294.2	605.5 ± 6815.1	0.91
HSP90a, ng/ml	104.2 ± 71.8	156.5 ± 106.3	0.004*
*Echocardiography*			
Proportion of abnormal segment, %	111 (64.9)	33 (63.5)	0.84
LVEF, %	54.3 ± 11.1	53.8 ± 11.4	0.75
Composite outcomes, n (%)	20 (11.6)	7 (13.5)	0.55
In-hospital death, n (%)	1 (0.6)	2 (3.8)	
Cardiogenic shock, n (%)	7 (4.1)	2 (3.8)	
Sustained VT/VF, n (%)	2 (1.2)	1 (1.9)	
Use of MCS, n (%)	14 (8.2)	3 (5.8)	

*Statistical analysis was done with Student’s t test or Chi-square test, where* p* < 0.05 was considered as significant

For clinical outcomes, there were 9 cases of cardiogenic shock, 16 cases with intra-aortic balloon pump (IABP) implantation, and 1 case of extracorporeal membrane oxygenation (ECMO) that needed cardio-pulmonary-resuscitation (CPR), and 3 cases of sustained VT/VF, 3 cases of in-hospital death. During the pandemic outbreak, there were 2 cases of cardiogenic shock, 2 cases with IABP implantation, 1 case of sustained VT/VF, 2 cases of in-hospital death and 1 case of ECMO that needed CPR. Among these, 1 case experienced both in- hospital death and use of ECMO. In contrast, in ACS patients before the pandemic outbreak, there were 7 cases of cardiogenic shock, 14 cases with IABP implantation, 2 cases of sustained VT/VF and use of MCS, and 1 case of in-hospital death. Among these, 1 case experienced both sustained VT/VF and the use of IABP implantation. In addition, 3 cases experienced both cardiogenic shock and the use of IABP implantation. Although more patients in the COVID19 group had tendency to have composite in-hospital complicated course or worse outcome (13.5%, 7/52) than that in the pre-COVID group(11.6%, 20/171), the difference between the two groups was not significant (*p* > 0.05) (Table 2).

### Logistic multi-factor regression analysis

Age/sex-adjusted models for identified history of smoking, HT, DM, exacerbation of symptoms, FMC within 2 h, CV, the pandemic, proportion of D2B within 90 min and admission methods were possible risk factors for the composite clinical outcomes in ACS patients. Logistic multi-factor regression analysis revealed that only age remained statistically significant risk factors for the composite clinical outcomes (HR = 1.3; 95% CI 1.12–1.50; *p* = 0.003). In the adjusted regression analysis, the pandemic was not associated with clinical outcomes (HR = 0.31; *p* = 0.053). Also, the FMC delay was put into multivariable logistical regression, the results remained unchanged (Table 3).

**Table 3:** The results of logistic multi-factor regression analysis

Variable	β	*p*
Age	1.26	0.009*
Gender	0.62	0.56
Time to worsen symptoms prior to entry	1.13	0.83
Previous MI	0.55	0.21
Proportion of FMC within 2 h	1.30	0.62
CV (LAD/LCX/RCA)	0.71	0.83
Group (COVID-19/pre-COVID-19)	0.40	0.06
Route of presentation (transfer or direct)	0.54	0.33
Proportion of D2B within 90 min	1.50	0.45

*Statistical analysis was done with binary logistic regression analysis, where* p* < 0.05 was considered as signifcant

## Discussions

There are 2 main findings from our retrospective study of archived data. First, there was a reduced patient admission during the pandemic, a significantly delayed FMC and a lower proportion of ACS patients with D2B within 90 min. Second, the proportion of transferred admissions, dual antiplatelet therapy (DAPT) and immediate thrombolysis increased during the pandemic. However, the pandemic has not been associated with worse outcomes under our adopted management.

In December 2019, an outbreak of pneumonia caused by a novel coronavirus in placing an unprecedented strain on patients, physicians and world healthcare systems that resulted in delay of treatment of patients with ACS and higher rate of in-hospital cardiac mortality [15, 16]. Thus, reduced admission, delayed FMC, and lower proportion of D2B within 90 min for ACS patients are understandable. During the pandemic, ACS patients tried to endure their symptoms until the chest pain was intolerable. This was also observed in several reports, stating a substantial drop in admissions, with a 48% reduction in the number of patients attending the emergency department with acute coronary syndromes (ACS) [5–9], longer FMC (318 vs. 82.5 h), average D2B (88 vs. 53 min, *p* = 0.033) and a reduction in the proportion of ACS patients within 90 min of D2B (71.4% vs. 80.9%, *p* = 0.042) in earlier studies [17–19]. We suppose this mainly being the result of standard COVID-19 infection verification before PCI and patient-related factors such as reluctance in seeking medical care [20] and fear of COVID-19 transmission in the hospital [18]. Therefore, finding a balance between risks related to untimely treatment of ACS patients and COVID19 infection control has become a global challenge during this pandemic.

Many strategies might be envisioned, and several have been already implemented [21–23]. In our study, worse outcomes did not differ between the two groups (OR = 1.3 confifidence interval, CI 1.1–1.5; *p* > 0.05) and the pandemic was not responsible for primary composite outcomes of in-hospital death, cardiogenic shock, sustained VT/VF and use of MCS in ACS patients. Based upon the management protocol for ACS used after Jan. 25, 2020 [11], our center provided a closer and more efficient collaborative team with regional non-PCI hospitals and advanced professional support through the social media-based platforms. Toušek P also found that modified strategies for invasive management of acute coronary syndrome during the COVID-19 pandemic did not cause an increase in hospital mortality [22]. Their modified treatment strategies were proposed by The European Association of Percutaneous Cardiovascular Interventions (EAPCI) and the Acute Cardiovascular Care Association (ACCA) for patients with ACS admitted to the hospital during the COVID-19 pandemic.

These data, relating to the outcomes of ACS patients, indicated that modified strategies were undoubtedly important and useful during the early phase of the pandemic.

Prompt recognition and early management are critical in reducing morbidity and mortality related to ACS [24], especially during the Covid-19 pandemic. Our adopted management emphasized a nearby treatment, safety, protection and multiple disciplinary team (MDT), and it was further established during the early pandemic through a regional STEMI care network. Under these guidelines, prehospital prompt emergency medical services (EMS) were activated through a social media- based platform during the early pandemic, since ischemic time duration is a major determinant of infarct size in patients with STEMI [24]. The plateform provided a venue for some excellent discussions and insight on prompt recognition, early management from MDT teams at institutions experiencing the effects of the pandemic and rapidly dispersed that prehospital information in order to better care for our patients. A meta-analysis of 10 case–control studies conducted by Barnes et al. demonstrated a two-fold increased risk of AMI in patients with recent influenza infection or respiratory tract infection, with a pooled odds ratio (OR) of 2.01 (95% CI 1.47–2.76) [14]. Therefore, quick decisions from the MDT were needed.

According to the Sichuan Provincial People’s Hospital proposed recommendations in China and following Peking Union Medical College Hospital recommendations, thrombolytic therapy was recommended over primary PCI if Covid-19 was confirmed or could not be excluded within a short time. Our study showed that a greater proportion of ACS patients received dual antiplatelet therapy during transport and immediate thrombolysis in prehospital emergency during the pandemic. Undoubtedly, thrombolytic therapy should not be the standard of care strategy and should be limited to particular situations, such as in non-PCI capable hospitals or when PCI cannot be performed within an acceptable time frame. However thrombolysis might be the best compromise for prompt reperfusion for the patient, buying time for a complete diagnosis to be made [25]. Moreover, a recent systematic review found that the administration of thrombolytic drugs, followed by immediate transfer to a PCI-capable hospital significantly decreased short-term mortality [26]. These patients might benefit from prehospital interventions to clinical outcome.

Because the new protocol emphasized nearby treatment, safety and protection, the pandemic was accompanied by a rise in the proportion of patients admitted to hospital from non- PCI facilities, which was not mentioned in recent study [6, 9]. Previous studies showed that there was an effect of transferred or direct admission on outcomes. There was a correlation between patients who were not transferred and increased comorbidity with much higher cardiovascular risk [27, 28]. This suggest that patients with AMI benefit from highly specialized services and interventions following interhospital transfer, which may partially account for favorable clinical outcomes.

Several limitations must be considered in this study, such as future studies with larger sample sizes, and longer follow-up periods. Firstly, since this study had a retrospective design, there might be some bias and heterogeneity between the two groups. Secondly, the sample size was relatively small and some important factors were not included, it may now represent the power needed to detect the potential related risk factors, more studies on the more important risk factors for the outcomes are therefore needed in the future. Finally, the long-term follow-up outcomes conducted were limited. Future research should investigate the long-term effect of the pandemic on the outcomes in ACS patients.

## Conclusions

The worst outcomes did not differ in occurrence between the two groups, which suggested that the newly adopted management strategies which emphasized nearby treatment, safety, protection with closer and more effective multiple collaborative team improved the clinical outcomes and provided optimal care for ACS patients during the early stage of COVID-19 pandemic.

## Data Availability

The datasets used or analyzed during the current study are available from the corresponding author on reasonable request.

## Abbreviations

ACS: Acute coronary syndrome
FMC: First medical contact
PCI: Percutaneous coronary intervention
VT: Ventricular tachycardia
VF: Ventricular fibrillation
MCS: Mechanical circulatory support
SARS-CoV-2: Severe acute respiratory syndrome due to coronavirus-2
AMI: Acute myocardial infarction
STEMI: ST elevation MI
NSTEMI: Non-ST elevation myocardial infarction
CSCAP: China STEMI Care Project
EMS: Emergency medical systems
ECG: Electrocardiograms
HscTn-I: High-sensitive cardiac troponin I
MDT: Multiple disciplinary team
CT: Computerized tomography
D2B: Door-to-balloon
CV: Culprit vessel
HSP90a: Heat-sensitive shock protein
BNP: B-type natriuretic peptide
TTE: Transthoracic echocardiography
LVEF: Left ventricular ejection fraction
SD: Standard deviation
IQR: Interquartile range
HT: Hypertension
DM: Diabetic mellitus
LMT: Left main trunk
LAD: Left ascending branch
RCA: Right coronary artery
LCX: Left circumflex artery
IABP: Intra-aortic balloon pump
ECMO: Extracorporeal membrane oxygenation
CPR: Cardio-pulmonary-resuscitation
DAPT: Dual antiplatelet therapy
